# A Novel Mechanomyography (MMG) Sensor Based on Piezo-Resistance Principle and with a Pyramidic Microarray

**DOI:** 10.3390/mi14101859

**Published:** 2023-09-28

**Authors:** Qize Fang, Shuchen Cao, Haotian Qin, Ruixue Yin, Wenjun Zhang, Hongbo Zhang

**Affiliations:** 1 School of Mechanical and Power Engineering, East China University of Science and Technology, Shanghai 200237, China; 2 Department of Mechanical Engineering, University of Saskatchewan, Saskatoon, SK S7N 5A9, Canada; chris.zhang@usask.ca

**Keywords:** mechanomyography, piezo-resistance, pyramidic microarray

## Abstract

Flexible piezoresistive sensors built by printing nanoparticles onto soft substrates are crucial for continuous health monitoring and wearable devices. In this study, a mechanomyography (MMG) sensor was developed using a flexible piezoresistive MMG signal sensor based on a pyramidal polydimethylsiloxane (PDMS) microarray sprayed with carbon nanotubes (CNTs). The experiment was conducted, and the results show that the sensitivity of the sensor can reach 0.4 kPa^−1^ in the measurement range of 0~1.5 kPa, and the correlation reached 96%. This has further implications for the possibility that muscle activation can be converted into mechanical movement. The integrity of the sensor in terms of its MMG signal acquisition was tested based on five subjects who were performing arm bending and arm extending movements. The results of this test were promising.

## 1. Introduction

Electromyography (EMG) is generated by electrical discharge of active motor units (MU) during muscle activation [1,2]. The action potential is generated by MU and then transmitted through the activation of Na and K ions on the muscle fibers [3], thus forming EMG. The EMG signal on the skin surface is called the surface electromyography (sEMG) signal [4]. Mechanomyography (MMG) is mechanical vibration induced by fiber contraction, which is caused by the release of calcium ions by the sarcoplasmic reticulum in the muscle fibers in response to an action potential [2,5,6]. Frangioni et al. found that the frog gastrocnemius muscles in a saline bath produced a single ringing sound with each force increment. The results of their studies indicate that the mechanism by which whole muscles produce and sustain low-frequency sound is primarily caused by transverse resonant vibrations [7]. sEMG and MMG are non-invasive compared with EMG, and MMG is not affected by changes in skin impedance caused by sweating compared with sEMG. Owing to its propagation through muscle tissue, the placement of MMG sensors is not required to be precise or specific [8]. MMG does not depend on its location in the muscle, whereas the normalized amplitude does and the centroid shifts with increasing force [9]. This further implies the possibility that muscle activation can be converted to mechanical movements. It has recently been generalized as a new sensor concept called a soft sensor [10,11].

Orizio et al. found that the MU global firing information (10–40 Hz range) may dominate the MMG spectrum, whereas the motor unit action potential (MUAP) shape information is mainly reflected in the EMG spectrum. MMG may be regarded, because of a partial fusion of the single events, as the summation of more or less distorted sinusoids and not as the summation of trains of fast distinct MUAP, as in the EMG [12]. This shows that MMG tends to be more sensitive than EMG to low-frequency vibrations of muscle activity, which is useful for examining muscle fatigue [13], strength [14], balance [15], and detecting small muscle movements [16,17]. MMG is highly related to muscle contraction and hand movements and can recognize muscle or hand patterns through linear discriminant analysis (LDA) classifiers with an accuracy of 89.7% [18,19].

Thin and flexible stretchable biosensors [20,21] are built by printing nanoparticles on soft substrates. They can be built as a type of skin on the human body, providing continuous monitoring of human states in a non-invasive and unobtrusive manner [22,23]. Flexible pressure sensors have been widely used for MMG recording of muscle contractions together with accelerometers (ACC) [24] and condenser microphones (MIC) [25]. A piezoresistive tactile sensor is a good choice for acquiring MMG signals [26,27,28]. Traditional piezoresistive materials, such as metal alloys and mixed silicon, usually have poor stretchability, nonlinearity, and large hysteresis [29], whereas nanoscale materials such as graphene, carbon nanotubes (CNTs), carbon black, MXene, metal oxides, and zinc oxide nanowires (ZnONW) have shown very high piezoresistivity [20,30,31,32,33,34,35]. Therefore, this study involved the use of nanomaterials and conductive polymers.

In this study reported in this paper, a novel MMG sensor was developed using a flexible piezoresistive MMG signal sensor based on a pyramidic PDMS microarray sprayed with CNTs. The experiment was conducted, and the results show that the sensitivity of the sensor can reach 0.4 kPa^−1^ in the measurement range of 0~1.5 kPa, and the correlation reaches 96%, which is unique compared to the 0.006 kPa^−1^ in the non-structured pressure sensor [36].

## 2. Material and Methods

### 2.1. Characteristics of CNTs and PDMS

CNTs have a high aspect ratio (average length/thickness) of 157.9 and good electrical conductivity of approximately 10^2^~10^6^ S/cm. The conductivity of CNTs depends on the nanoparticle pathways. When external pressure stimuli or temperature stimuli are applied to CNTs, the pathways change, and thus the conductivity changes [21]. More specifically, piezoresistivity is primarily attributed to the variation in tunneling resistance when the interfiller distance changes under external stress. External pressure induces stress concentrations at small contact spots and local deformation in the microstructure. Consequently, the contact area between the interlocked microarrays increased significantly and affected the tunneling resistance at the contact spots. The result is a large tunneling piezoresistance in flexible films [37].

Based on the theory of piezoresistive sensors discussed above, the compressibility of a material that is in direct contact with the applied force is important. The compressibility of the material used should be high so that a small applied force can significantly affect tunneling resistance. A low mechanical modulus is required to achieve a high mechanical sensitivity. Elastomers, such as PDMS (Sylgard 184, Dow Corning, Wiesbaden, Germany), typically have a modulus of ~2 MPa with good compressibility, making them a good choice for sensor base layers [38].

### 2.2. Simulation of Array Sensor Structure

**Figure 1:**
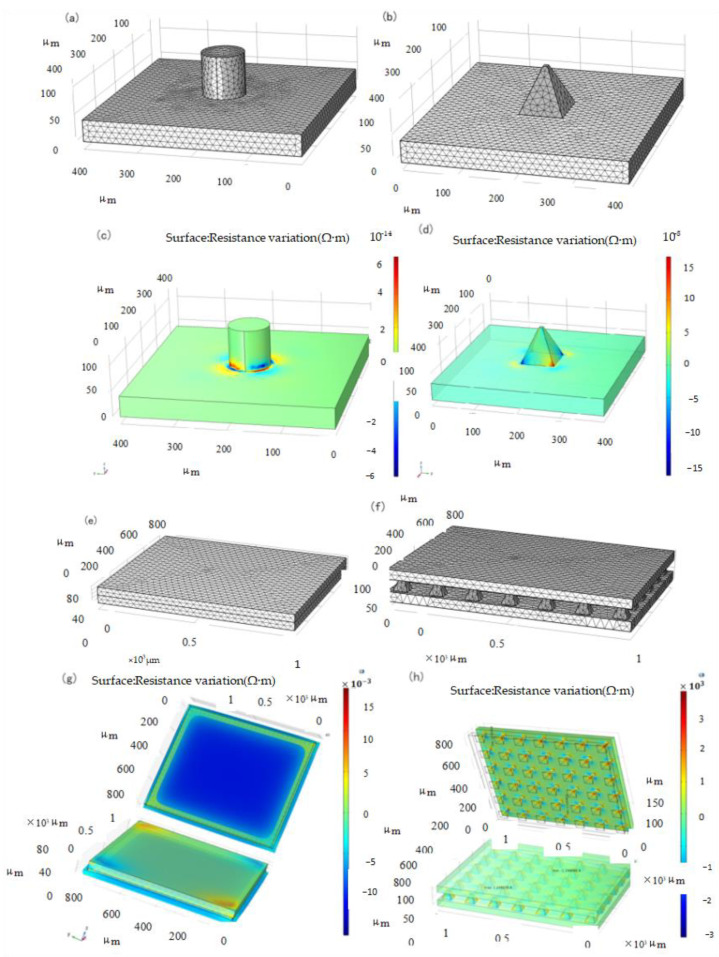
Meshing and Resistivity Change Simulation. (**a**) Cylindrical structure, (**b**) Pyramid structure, (**c**) Cylindrical structure, (**d**) Pyramid structure, (**e**) Non-structure, (**f**) Pyramid structure, (**g**) Non-structure, (**h**) Pyramid structure.

An important factor is the support structure upon which the film is built, particularly the contact area [30]. Several support structures, such as hemispheres, pyramids, and pillars, have been used to build piezoresistive film sensors [21]. To obtain the optimal microstructure, the Multiphysics module in COMSOL was used to conduct the approximate and discrete processing of the microstructure model to study the dynamic response of the elastic body under pressure. The parameters of the layered materials are listed in Table 1. After the corresponding constraints are set and the terminals and grounding are imposed, the structure is discretized. Owing to the small size of the microstructure, the precision of the size of the partition unit is designed to be ultrafine, and its grid pattern is shown in Figure 1a,b.

As shown in Figure 1c,d, the simulation resistance variation diagram of the two structures under 1 kPa load, respectively, shows that the resistance layer of the pyramid structure has a larger variation range than that of the cylindrical structure when the stress is the same; therefore, the pyramid structure resistance layer structure is adopted to improve the sensitivity. To verify the sensitivity of the microstructure sensor, an overall simulation of the sensor was performed. The constraint conditions of the two simulations were set to be the same as those of the materials and related parameters, and the unit division was also adopted with the same precision, as shown in Figure 1e,f.

Tee et al. found that the greater the angle of the slope defined by the sidewall perpendicular to the base sidewall, the greater is the sensitivity of the geometrical shape to compression [38]. Our simulation can also be explained by considering the stress distributions for different geometrical shapes. In a cylindrical structure, the stress distribution is fairly constant throughout the height of the cylinder owing to its uniform cross-sectional area. However, when the shape is pyramidal, the stress distribution is non-uniform and concentrated at the pointed tips because of the smaller contact area rather than the broad base of the pyramid structure. Thus, the pyramid tips compressed more, resulting in higher mechanical deformations at the top [38]. This results in a large tunneling piezoresistance in the piezoresistive layer, which makes the pyramid structure more sensitive. Considering the pressure-sensing capabilities of single-sided microstructured composites, interlocked composites with double microstructured surfaces are expected to further enhance the pressure sensitivity owing to the larger pressure-induced deformation of the interlocked microstructures [30].

The simulation of the piezoresistive change with or without the microstructure is shown in Figure 1g,h. The pressure application magnitude and direction of the two sensors were applied from the upper plane. It can be seen from the analysis results that the resistance change of the sensor without microstructure is of the order of magnitude of $10^{-3}\;\Omega \cdot m$, and that of the sensor with pyramid microstructure is of the order of magnitude of $10^{3}\;\Omega \cdot m$. Therefore, in the same situation, sensors with pyramid microstructures can produce obvious resistivity changes at smaller forces, thereby improving sensitivity.

## 3. Experimental Section

### 3.1. Design of Piezo-Resistive Array Sensor

**Figure 2:**
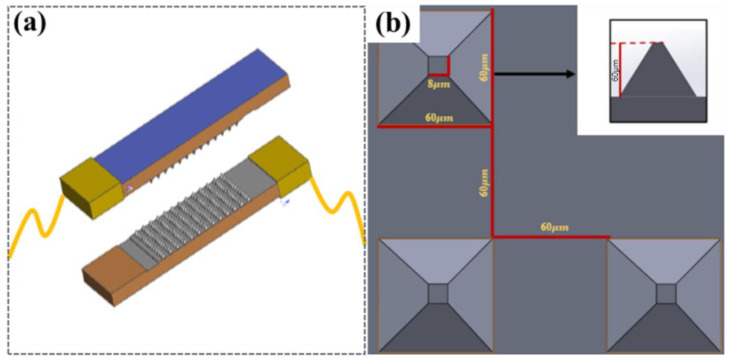
The concept design of the MMG sensor. (**a**) Schematic diagram of the sensor, (**b**) Schematic diagram of the pyramid structure.

The piezoresistive sensor design was mainly divided into upper and lower substrates, with piezoresistive and conductive layers in the middle. Copper foil was used as an external conductor in the two sections, and CNTs were not sprayed on the other bottom layer corresponding to the copper foil to avoid direct conduction of the microstructure layer in the upper and lower layers, as shown in Figure 2a. PDMS was used as the base layer of the sensor owing to its biocompatibility and printability [39], CNTs were used as the conductive material, and a viscous copper foil lead wire was used in the upper and lower sections. The overall size of the sensor was 25 mm × 12 mm, and its thickness was 2 mm.

Considering that the shape and resistance change in different microstructures of sensors under the same load were not the same, two types of microstructures were designed: a cylinder (height to diameter ratio of 1:1) and a pyramid structure with a square bottom surface (ratio of high bottom edge is 1:1). As shown in Figure 2b, a piezoresistive simulation of the sensor was performed.

### 3.2. Fabrication of Piezo-Resistive Array Sensor

There are many manufacturing methods for silicon templates to produce microstructure molds, which can be divided into two-photon printing, femtosecond laser printing, silicon plate etching, 3D printing, and so on according to different precision requirements. Considering the existing conditions of the laboratory and the cost effectiveness, under the condition of meeting the requirements of the microstructure processing accuracy of the sensor, we adopted a 3D printing template and selected B9R-3-Emerald Resin(B9Creations) as the printing material owing to its promising results [32]. The follow-up steps were as follows. Prior to using a 375 nm UV lamp for photocuring, the printed mold was rinsed with anhydrous ethanol (≥99.7%) to remove residual burrs. A demolding agent (GW-8500, DAIKIN, Japan) was sprayed using a rotating nozzle. Subsequently, the printed mold was placed in a drying box at 40 °C, dried for 4 h, and then removed and allowed to use.

**Figure 3:**
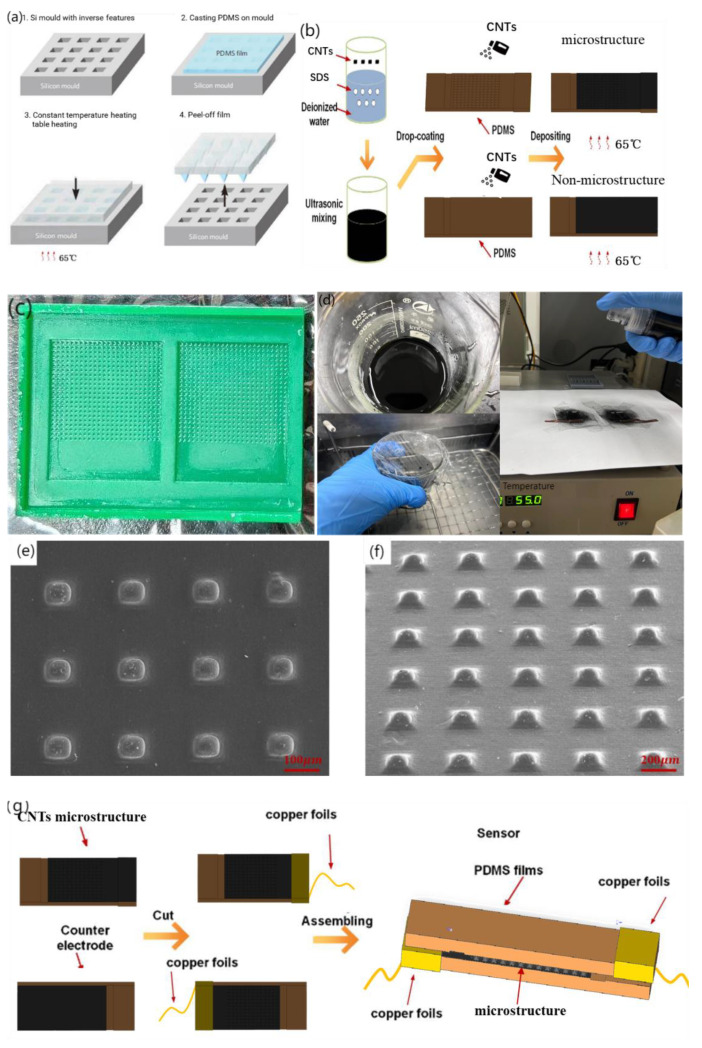

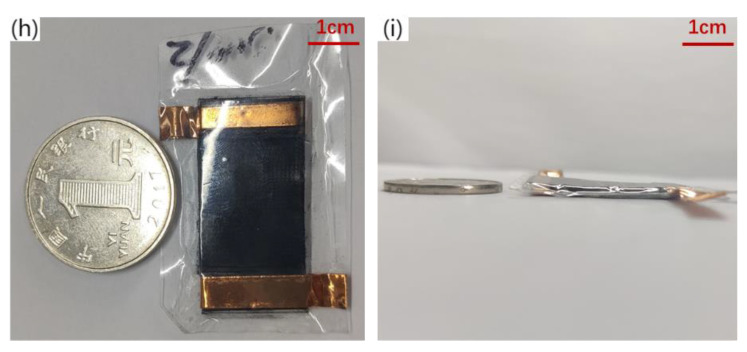
Fabrication technology of piezo-resistive array sensor. (**a**) PDMS substrate over-molding process, (**b**) Fabrication of piezo-resistive Layers for Flexible Pressure Sensor, (**c**) Authentic image of 3D-printed mold, (**d**) Authentic image of experiment steps, (**e**) 90°viewing angle SEM image of microstructure, (**f**) 45°viewing angle SEM image of microstructure, (**g**) Flexible piezo-resistive Sensor Assembly, (**h**) front view Physical map of flexible sensor, the ruler uses one yuan coin in China (**i**) side view Physical map of flexible sensor.

Common materials with elastic and solid properties for the preparation of the sensor base layer include metal foil and organic polymers, among which polymers primarily include polydimethylsiloxane (PDMS), polyimide (PI), polyparaxylene (PX), and polyethylene film (PET). PDMS exhibits a good balance between stretchability, processing difficulty, and biocompatibility. The substrate of the flexible sensor was thermomolded with PDMS (Sylgard 184, Dow Corning, Wiesbaden, Germany) to construct an MMG sensor with a pyramid microstructure and flexible substrate, as shown in Figure 3a. A polydimethylsiloxane (PDMS) prepolymer base solution and curing agent were poured into the plastic cup at the same time (mass ratio of 10:1) and fully stirred until a uniform mixture was obtained, and the remaining bubbles in the mixture were removed in the empty extraction chamber. Subsequently, the vacuumed PDMS was evenly coated on the processed silicon mold, and the gas between the template microstructure and PDMS was extracted in the vacuum machine again until no bubbles emerged on the PDMS surface. After gas extraction, the mold bearing PDMS was placed on a constant-temperature heating table after balance debugging and stood at 65 °C for 4~5 h. After the mold was removed from the heating table and restored to room temperature, PDMS was slowly removed from the mold for further use.

Common conductive materials that can be used as resistance layers include carbon nanotubes (CNTs), graphene, nanomaterials, gold, silver, and copper. Carbon nanotubes have a high surface area, high stability, and low cost because of their internal voids, making them ideal conductive materials for the resistance layer of flexible sensors. For an experimental comparison, sensors with and without microstructures are fabricated in this section. Figure 3b shows the sensor preparation process. Figure 3c,d) show the authentic images of the 3D-printed mold and the experimental steps, respectively.

After peeling, 500 mg of CNTs was weighed using an electronic balance and poured into 40 mL deionized water for ultrasonic shock for 30 min. Subsequently, 150 mg of sodium dodecyl sulfate (SDS) was added to the mixed solution as a surfactant. After ultrasonic treatment for 30 min, CNTs were fully dispersed. An ultrasonic probe was used for 30 min, and after standing, a pipette was used to collect the supernatant for later use. Subsequently, the PDMS base with the microstructure was placed into the plasma cleaner upward, so that the pressure gauge pointer of the pressure-reducing valve was between 0.1~0.2 MPa. The substrate was cleaned at 600 and 800 V for 2 min to improve the hydrophilicity of the surface and facilitate the attachment of the CNTs dispersion liquid. The treated substrate was placed on a constant temperature heating table at 80 °C, and 15 mL of CNT-dispersed liquid was placed in the spray gun. The dispersed liquid in the spray gun was evenly sprayed onto the substrate surface with a microstructure three times (5 mL each) at an interval of 15 min. After spraying, the sensor was left to stand for 20 min and then removed. A scanning electron microscopy (SEM) image of the sensor surface after spraying is shown in Figure 3e,f.

The assembly process of the flexible piezoresistive sensor is illustrated in Figure 3g. After cutting the excess part of the base after mold turning, the heresy part without the microstructure of the two bases was bound with a viscous copper foil. CNTs were not sprayed on the corresponding bottom layer of the copper foil to prevent the copper foil from directly conducting with the bottom CNTs. This results in a conductive path that does not pass through the resistance layer with a pyramidal microarray with a large resistance. The copper foil we used was self-adhesive and could be attached to the CNTs on the substrates. It has low surface oxygen characteristics and excellent conductivity and is mainly used in electromagnetic shielding and antistatics. CNTs are hydrophilic materials; therefore, humidity is an important physical quantity that affects the performance of the sensor [40]. When we encapsulated two substrates, 20 um PDMS film and dielectric elastomer film (800 um) were used to seal it to isolate the influence of humidity on the sensor. The physical diagrams are shown in Figure 3h,i. The upper and lower substrates provide the substrate support for the sprayed resistance layer and determine the path of conduction, which is formed by the current through the copper wire, copper foil, CNTs on the substrate, and CNTs on the pyramidal microarray. Because of spraying, the thickness of the substrate has certain requirements, and too thin will cause the spray layer to fall off with bending.

### 3.3. Flexible Sensor Characterization

**Figure 4:**
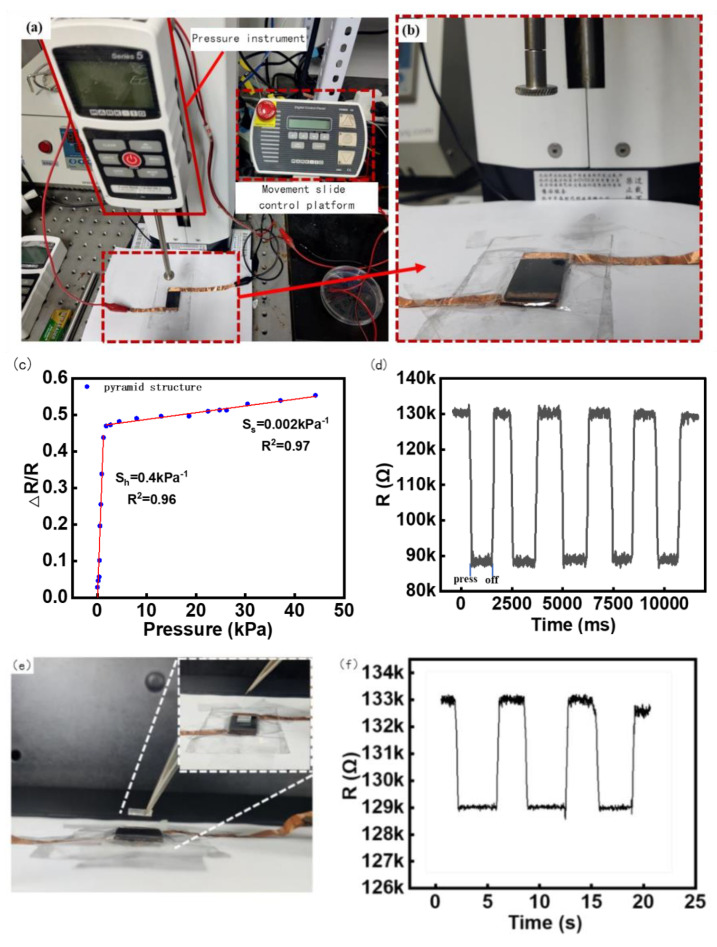
Sensor Test Platform and Experiment Results. (**a**) Motorized Tension/Compression Test Stand (ESM303, Mark−10, Beijing Jipin times Technology Co., Ltd), (**b**) Pressure test details display, (**c**) The relationship between sensor resistance change rate and pressure, (**d**) Sensor Repeatability Test, (**e**) Minimum response stress test Experimental plot, (**f**) Minimum response stress test Data plot.

The sensitivity of the manufactured flexible sensor was tested using mechanical instruments; the test environment is shown in Figure 4. Figure 4a shows the entire test mechanics system, including the tension and pressure analyzer, motion control platform, and test bench. Figure 4b shows a schematic of the test process for the contact head and sensor. 

The sensor was fixed to a mechanical tester, and the applied force range was 0~12 N.

The pressure increment was 0.02 N downward each time through the press, and the time of each applied force was maintained at 3 s. The advance distance was set as 0.01 mm/ time through a circular contact radius of 4 mm, and the resistance and change with time were collected by the electrochemical analyzer. The final sensitivity test results are shown in Figure 4c. As can be seen in Figure 4c, when the sensor sensitivity is about 0–1.5 kpa, the deformation resistance of the microstructure of the resistance layer is small when the sensor is stressed, so that the contact area of the upper and lower conductive layers becomes larger, the resistivity changes increase, and the sensitivity is approximately 0.4 kPa^−1^. In the low-pressure range of 0 to 1.5 kPa, it can effectively detect pressure from a variety of sources, including sound waves, light objects, and finger touch forces, which is perfectly suitable for our detection of sound vibrations below 50 Hz [41,42]. Notably, it can be found that the resistance changes by about 0.4 times the original resistance even under the tiny pressure produced by the sound of approximately 2–20 Pa, indicating the high sensitivity of the sensors [41]. When the pressure exceeds 1.5 kPa, the deformation resistance of the sensor microstructure increases owing to the increase in the intermolecular force, the deformation of the microstructure and the substrate becomes small, the change in resistivity is weak, and the sensitivity in the stable stage is approximately 0.002 kPa^−1^. 

A load of 2.5 kPa was repeatedly applied and unloaded on the sensor 1000 times, and the resistance change data of the response were collected. According to the calculation in Figure 4d, it can be seen that the range of resistance change was close to the same value each time, and the repeatability of the sensor was relatively stable.

The minimum reaction force was the minimum force that could be detected by the sensor. Because the minimum increment of the tester is 0.02 N, which cannot meet the requirements, square PDMS with an elastic side length of 7 mm weighing 80 mg was used as the pressure object in this experiment, and the gravity was approximately 0.0008 N. The PDMS weight was held using tweezers and slowly placed on the sensor surface, as shown in Figure 4e. After the data stabilized, the weight was removed using tweezers, and the resistance change was recorded three times, as shown in Figure 4f. It can be seen that three consecutive lifts and places of heavy objects should be able to make the resistance of the sensor change significantly and reproducibly. Combined with Figure 4d, the excellent responsiveness of the sensor in the face of a small force was reflected.

## 4. Results and Discussion

The MMG signal acquisition test was performed using a flexible piezoresistive array sensor. The sensor and flexible circuit board were packaged, a system performance test was carried out, and the MMG signal and its threshold were analyzed.

### 4.1. Design of MMG Signal Conditioning Circuit

**Figure 5:**
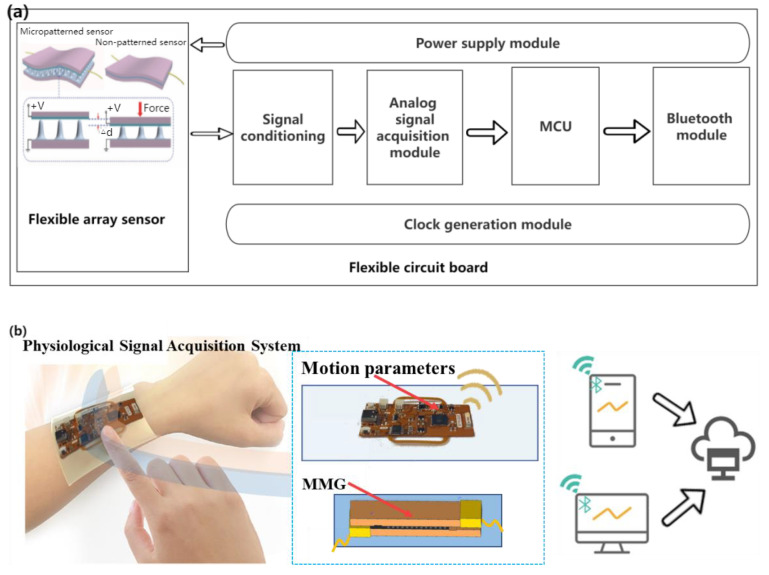
(**a**) Block diagram of hardware design scheme of acquisition system, (**b**) Flexible wearable multi-mode physiological signal acquisition system.

Through the analysis of the design requirements, on the premise of meeting the non-invasive, flexible wearable, multi-mode signal acquisition, data transmission, and other functions, a general block diagram of the hardware part is shown in Figure 5a. A flexible wearable multimode physiological signal acquisition system is shown in Figure 5b. The control electronics were designed as a flexible printed circuit (FPC, polyimide substrate) board (Supplementary Figure S1 and Supplementary Table S1) for analog signal acquisition and Bluetooth communication. The ESP32-PICO-D4 chip (Lexin Technology, Shenzhen, China) was selected as the control and communication chip owing to its small size, powerful function, low energy consumption, and low price. The signal conditioning module is the key to the hardware part of the acquisition system, which generally includes signal processing functions, such as program-controlled amplification and filtering.

**Figure 6:**
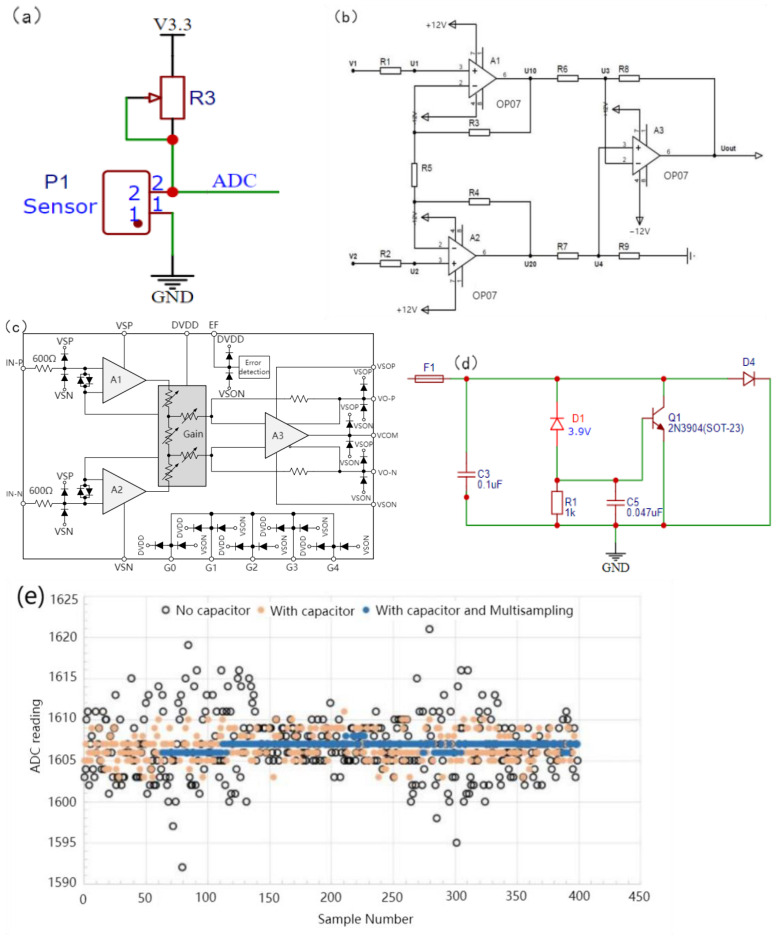
Design of MMG signal conditioning circuit. (**a**) Current to voltage divider circuit, (**b**) Differential amplifier circuit, (**c**) Equivalent Internal Circuitry, (**d**) Input protection circuit, (**e**) Capacitance and Multiple Sampling Noise Suppression.

The current signal in the 4~20 mA input corresponds to a linear voltage output and has no correlation with the size of the resistance; therefore, it is necessary to control the size of the input current before use. The voltages at both ends of the piezoresistive MMG signal sensor were collected, and the resistance of the sensor was related to the MMG signal of the skin and face. To reasonably control the voltage within a range of changes and reduce resistance noise, an adjustable resistor (50 Ω~1 MΩ, ±20%) was selected in series to balance the initial voltage at both ends of the sensor. The current-to-voltage divider circuit is shown in Figure 6a. After the signal is processed by the voltage divider circuit, the current signal becomes weak, which is quite different from the ideal input signal range. ESP32 has a built-in 12 bit ADC, its precision is 244 μV. When the unamplified voltage change was less than this value, the sampling chip produced an acquisition error. If the signal is small, the impact of the noise increases. Therefore, this circuit must amplify the voltage signal to reach the ADC sampling input range (0~5 V). To reduce the common-mode input signal, suppress zero drift, and reduce noise and interference, a differential amplification circuit was selected for this device, as shown in Figure 6b. The differential amplifier circuit, composed of resistors and common amplifiers, has a constant amplification factor, which is difficult to debug in the later stage. A programmable AD8555ARZ (Analog Devices, Inc., Wilmington, MA, USA) operational amplifier was adopted, whose equivalent internal circuitry diagram is shown in Figure 6c. This connection can be found in the analog data acquisition module (Supplementary Figure S2).

The full range of the ADC voltage acquisition of the ESP32 is related to the attenuation ratio. When the attenuation ratio is 11 dB, the full range is 3.9 V, and the 3.3 V voltage provided by the voltage conversion module is adopted to supply power to the entire piezoresistive sensor. When the series voltage divider resistance was close to infinity relative to the sensor resistance, the piezoresistive sensor was close to the divider full voltage. After amplification, the analog voltage definitely exceeds the acquisition range of the ADC and then damages the entire device. Therefore, an input-protection circuit is crucial. The protective circuit of the device is shown in Figure 6d. When the voltage reaches the Zener voltage of 3.9 V, D1 breaks down, and the gate level of Q1 is subject to the Zener voltage. After Q1 is turned on, the input voltage is grounded so that the fuse is blown to achieve the effect of the open circuit. 0.047 uF capacitor is used as the protective capacitor of thyristor Q1 to weaken the steepness of the voltage peak of the switch and prevent the wrong touch of the thyristor. It is very important to select the capacitance value, which can filter noise and will not increase the delay of thyristor conduction after the pulse because of the high capacitance value, which causes the analog-to-digital converter of ESP32 to have low-resolution measurements. In practice, bypass capacitors (for example, 100 nF ceramic capacitors) can be connected to pads corresponding to analog pins, or multiple sampling can be used to further mitigate the effects of noise. The 100 nF bypass capacitors we used can be found in the analog data acquisition module, whose identification is C12 (Supplementary Figure S2). It can not only filter out the noise interference of the power supply but can also be used as a decoupling capacitor to prevent fluctuations in the integrated circuit from coupling to the power supply. Figure 6e. shows the interference suppression using capacitors and multiple samplings. The method “with capacitor multisampling” increases sampling rate at the same sampling frequency compared with the method “with capacitor” on the ESP32 program, which means that the ADC can collect more signal points at a time, it could reduce the power of ADC quantization noise in the effective frequency band.

Considering the multiple physiological signal acquisition requirements of this device, in order to facilitate secondary development during the AD/DA acquisition and conversion, the acquisition channel with MMG signal, pulse signal, and other analog signals is designed. The noise of signal conditioning mainly includes power resistance noise $e_{NR}$, current noise $e_{NI}$, voltage noise of operational amplifier $e_{NV}$, and root mean square error of signal conditioning noise $e_{NAvg}$, which can be obtained by [43]

(1) $$e_{NAvg}=\sqrt{e_{NR}^{2}+e_{NI}^{2}+e_{NV}^{2}}$$

Resistance noise is related to the material properties of the resistance itself. The calculation of the power supply resistance noise $e_{NR}$ was obtained by [44]

(2) $$e_{NR}=\sqrt{4kT_{k}R_{eq}df}$$

In Equation (2), $k=1.38\times 10^{-23}J/K$, $T_{k}$ is the thermodynamic temperature, $R_{eq}$ is the equivalent electrical impedance, df is the equivalent frequency differential.

The calculation of the operational amplifier voltage noise $e_{NV}$ is obtained by [43]

(3) $$e_{NV}=\sqrt{e_{NBB}^{2}+e_{NI}^{2}}$$

where $e_{NBB}$ can be calculated using Equation (4), and $e_{BB}$ is the noise bandwidth. From Equation (3), it can be seen that the operational amplifier voltage noise $e_{NV}$ is mainly related to flicker noise $e_{NBB}$ and equivalent input noise voltage, both of which are related to the reciprocal of the frequency f.

(4) $$e_{NBB}=e_{BB}\sqrt{df}$$

Current noise $e_{NI}$ can be converted into the operational amplification noise by

(5) $$e_{NI}=R_{eq}I_{n}$$

where $I_{n}$ is the equivalent current and $R_{eq}$ is the equivalent resistance.

According to the common programmable amplifier key indicators in Table 2, the maximum value of each key indicator was selected to estimate the noise peak. When the amplification gain is 128, the noise calculation of the conditioning circuit is carried into Equations (2)–(4) to obtain

(6) $$\begin{array}{cc} e_{NAvg1} & =\sqrt{{\left(1308nV\right)}^{2}+{\left(420nV\right)}^{2}+{\left(22nV/\sqrt{Hz}\times \sqrt{31Hz}\right)}^{2}}\\ & =1.374\mu V \end{array}$$

When the multiple of the selected index parameter is four, the total relative error of the conditioning circuit is calculated as:

(7) $$E_{Nsgc}=\frac{32\times eN_{Avg}+e_{NAvg1}}{3.3V}$$

$E_{Nsgc}=0.0017\%$, which meets the design requirements of $E_{Nsgc}<0.0035\%$.

### 4.2. Biceps and Gastrocnemius MMG Signal Tests

**Figure 7:**
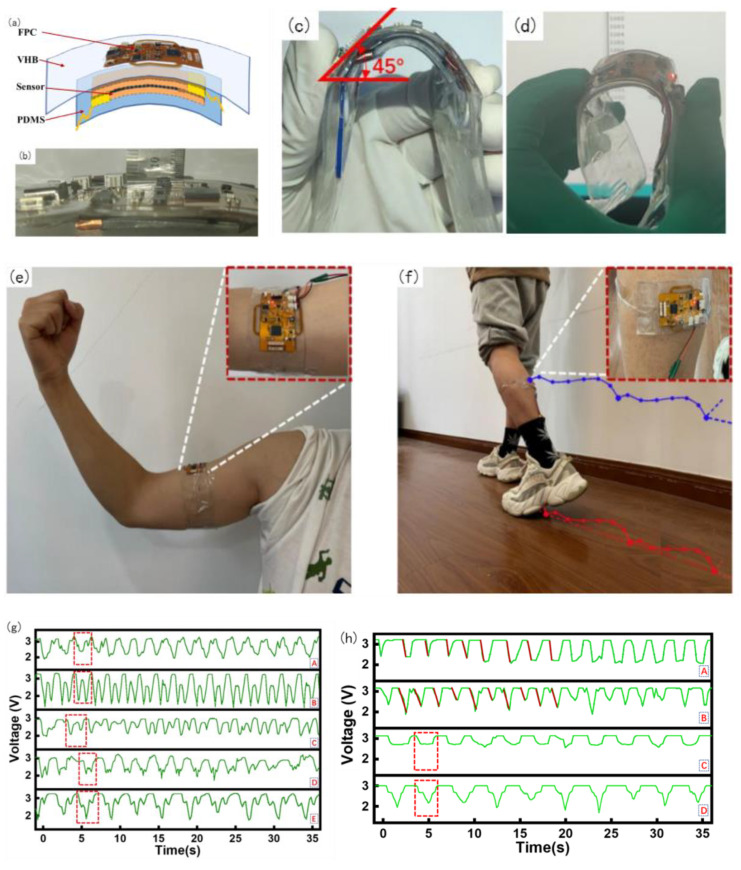

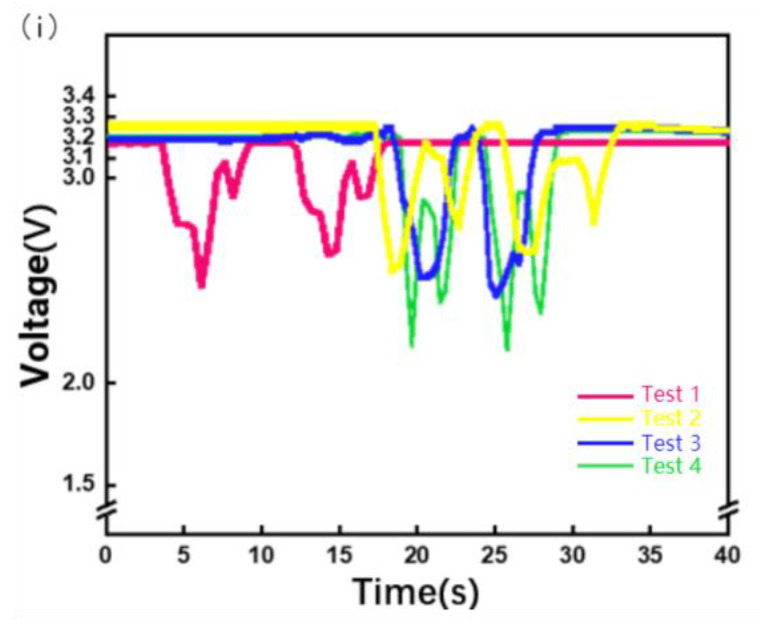
Wearable device package, Physical test and MMG signal test diagram. (**a**) Wearable device package diagram, (**b**) device thickness measurement, (**c**) bending test, (**d**) working bending test, (**e**) biceps wearing test, (**f**) gastrocnemius wearing test, (**g**) Biceps muscle MMG signal test, (**h**) Gastrocnemius muscle signal test, (**i**) Raw data of MMG of four times wearing.

The entire device was divided into four layers: the top layer was an FPC, mainly for signal acquisition and processing, the second layer was an elastic dielectric, the third layer was a flexible sensor, and the fourth layer was a PDMS film. To facilitate wear and meet the ductility and recoverability requirements, the packaging of this device adopts the dielectric elastomer 3 M VHB (1 mm), which is sticky, malleable, and resilient. The FPC acquisition device can be tightly fitted with a flexible sensor based on the viscosity of VHB and can be bent along with the sensor and acquisition device. The sticky contact with the skin affects the state of the skin, thus affecting the accuracy of the MMG signal acquisition, and it is obviously invasive to wear for a long time. 100 um PDMS film was selected and plasma-treated to increase its adhesive viscosity with VHB. The entire device was used for the encapsulation of the measurement layer, as shown in Figure 7a.

The thickness of the packaged physical object is shown in Figure 7b; the thickness of the entire device was 8 mm. After testing, normal bending can reach 45° or even larger, as shown in Figure 7c. Under the condition of 45° bending, a power-on bending test was performed after the code was burned. During the entire process, the device was powered using Type C. It can be observed that the communication was still normal under the condition of 45° bending, as shown in Figure 7d.

After obtaining approval from the Ethics Committee of East China University of Science and Technology, under the premise of adhering to the Declaration of Helsinki, a total of five subjects (four males and one female) were recruited for this experiment, aged from 23 to 28 years old, including four males and one female. In the first group of acquisition experiments, physiological signal acquisition devices were used in the biceps brachii to collect MMG signals by performing arm bending and arm extension movements, as shown in Figure 7e. Castillo et al. emphasized the importance of the contact force between the sensor and skin in the evaluation of MMG activity and found that the classification performance tends to increase as the contact pressure increases [45]. However, Posatskiy found that MMG recordings were highly susceptible to contamination from limb movements [46]. Therefore, the MMG sensor in our experiment was attached to the muscle belly to reduce the influence of the force generated by the limb movement on the muscle vibration, namely the MMG signal detection. In the second group of experiments, the same subjects performed different movements to collect MMG signals of the same part by attaching the acquisition device to the stomach of the gastrocnemius muscle of the lower extremities. The test wearing device is shown in Figure 7f, where 3.7 V lithium battery is used for power supply.

The main purpose of this device is to check the integrity of the MMG signal acquisition and observe the regularity of the signal during movement for the subsequent analysis and processing application of the MMG signal. The MMG data for the five subjects are shown in Figure 7g. Among them, five subjects performed arm bending as uniformly as possible within their abilities, and the five curves reflected the information of each subject very well.

Because the collection of this device uses the series resistance partial voltage to form the association of the MMG signal, the intensity of the MMG signal has an opposite trend to the measured value of the voltage. The B curve in Figure 7g shows that the subject often worked out, had low body fat, and strong muscle strength. Within one arm flexion and extension movement cycle, there are two troughs in the muscle signal. This is because the muscle pattern of B is obvious, and the biceps brachii moves up from under the skin during the arm flexion process. During arm extension, the biceps brachii muscle is blocky and returns to its original state. It can be observed that the MMG signal in B is stable and regular. Subjects C and D exercised less and had two valleys in the MMG signal during a test cycle; however, during arm bending and extension, the symmetry was lower, the MMG amplitude was lower, and the muscle strength was weaker. In subjects A and C, there was only one trough in the waveform, and no trough in the process of arm extension. This was because muscle fibers from the biceps did not form. There was no direct dispersion of muscle fibers and no muscle retraction during arm extension. Based on the above summary, the conditions for judging the severity of the MMG signal can be determined by the bottom value of the MMG signal: the lower the bottom value, the higher the muscle activity. Summarizing the analysis of biceps muscle MMG signals in five subjects, the symmetry and regularity of the MMG signal waveform are related to muscle strength, which is related to a person’s body fat, BMI, etc.

In the second group of tests, the belly of the gastrocnemius muscle with the acquisition device placed on the lower limb was drawn out, as shown in Figure 7f. The following actions were performed separately. (A) Keep the lower leg motionless and lift the foot; (B) hold the leg in place and pull the leg back during the leg lift; (C) squat with the thigh 90° perpendicular to the calf as far as possible; (D) take the knee as the axis and squat with the thigh greater than 90°. The processed data from the MMG signal acquisition experiment are shown in Figure 7h. As shown in Figure 7h, in the process of action A, the MMG signal action relative to action B has a comparatively severe reaction degree. This is because the thigh is not the main force output in the process of lifting the foot, mainly the calf gastrocnemius plays a main role, and B action is mainly the leg thigh quadriceps and gastrocnemius muscle exert force, so the gastrocnemius muscle load is shared, and the MMG signal change is not so steep.

Action C is mainly in the process of 90° squat; gastrocnemius muscle change is relatively stable; when it reaches approximately 90°, the muscle signal is at the bottom, after standing back to the initial state. Action D relative to action C, in the process of falling and rising of the MMG signal, will have a secondary decline and the second rise, because when the thighs and legs reach 90° at the bottom for the first time, when more than 90° continues to squat, the thighs and legs will contact at the knee, and muscles produce an extrusion interaction, which increases the scope of the vibration of the muscle fibers. The force of muscle retraction increased, and a secondary descent occurred. In the recovery stand, thigh muscles and calf muscles from first contact, the compression strength drops, producing a rise, and the second is by the stress state of the gastrocnemius muscle recovery, resulting in a second.

### 4.3. Error Analysis of Muscle Signal

Because this device is a flexible piezoresistive sensor that fits the skin, owing to the weight of the device itself and slight preloading force will be applied in the process of wear, the size of preloading is different because the wearing position is not necessarily the same each time, or because of the difference in users, so the initial resistance value of the piezoresistive sensor is different, so the initial voltage is different. Therefore, setting a fixed initial voltage affects the evaluation of MMG signals. The initial voltages of the four dressings are shown in Figure 7i. As shown in Figure 7i, the starting voltage of the MMG signal cannot be calibrated, leading to a starting error. Therefore, subsequent research needs to use machine learning algorithms to classify and analyze the percentage change in the MMG signal, slope of the MMG signal during the action, peak change in the MMG signal, fatigue of the muscle, and action.

## 5. Conclusions

In this study, we developed a flexible piezoresistive MMG signal sensor based on the piezo-resistivity principle and pyramidal PDMS microarray sprayed with CNTs. The sensor has a high sensitivity (0.002 kPa^−1^) in comparison with existing approaches, such as accelerometers and microphones. The sensor is highly robust and resilient to the fitness of a rigid sensor, skin moment, and noise in the skin environment [47,48]. The microneedle sensor device was plasma-processed and encapsulated with VHB, and its responsiveness to a small force was approximately 2 Pa. The thickness of the entire device was approximately 8 mm, which met the requirements for wearable comfort and degree of fit. By testing the MMG signal, the sensitivity of the microarray sensor can reach 0.4 kPa^−1^ in the measurement range of 0~1.5 kPa, and the correlation reaches 96%. In the working state, the bending degree can reach 45°, the wireless communication distance can reach 5 m, and the MMG signal test can observe obvious actions and individual differences. In the flexion and extension test, when the movement frequency was lower than 2 Hz, the judgment of logic OR only for the biceps flexion test reached 100%. Finally, the sensor is highly manufacturable. In the future, we plan to develop soft sensors based on the generalization of the concept of MMG for point-of-care devices for early diagnosis [49].

## Figures and Tables

**Table 1:** The materials parameter setting of COMSOL simulating.

The Structure Layer	Material	Thickness (μm)	Young’s Modulus (MPa)	Poisson’s Ratio (nμ)
Base layer	PDMS	50	2400	0.4
piezo-resistive layer	PDMS/CNTs	5	1.2	0.5

**Table 2:** Common Programmable Amplifier Key Indicators.

Index Parameters	Work Environment	Index Number	Unit
Voltage noise (RTI)	f = 0.01~10 Hz, G = 128 f = 1 kHz, G = 128	420 22	nV $nV/\sqrt{Hz}$
Current noise (RTI)	f = 0.01~10 Hz, G = 128 f = 1 kHz, G = 128	1.7 90	pA $fA/\sqrt{Hz}$
Input offset voltage	All gain	±(5 + 45/G)	μV
Input impedance	Single-ended and differential	>1	GΩ
Nonlinearity	unloaded	1.5	ppm

## Data Availability

Data is contained within the article or Supplementary Material.

## Supplementary Materials

The following supporting information can be downloaded at: https://www.mdpi.com/article/10.3390/mi14101859/s1, Figure S1: Layouts of the FPC board. (a) Layouts of the FPC board with coppering (b) Layouts of the FPC board without coppering (c) Photos of the FPC board with key components (Supplementary Table S1) labeled (d)Photos of power supply module connecting with FPC board; Figure S2: Schematic connections of the analog data acquisition module and bluetooth data transmission module. The analog data acquisition module consists of the AD8555ARZ. The bluetooth data acquisition module consists of a microcontroller (MCU, ESP32-PICO-D4) with on-chip analog-to-digital converter (ADC) and on-chip bluetooth module. Table S1: Key components used in the control electronics. All of the components are commercially off the shelf.
