# Supplementary material for: A Novel Mechanomyography (MMG) Sensor Based on Piezo-Resistance Principle and with a Pyramidic Microarray
Source: Micromachines (Basel). 2023 Sep 28;14(10):1859. doi: 10.3390/mi14101859 (PMC10609147; doi:10.3390/mi14101859)
Supplement: Supplementary file 1 [file micromachines-14-01859-s001.zip › micromachines-2569009-supplementary.pdf]

(a)

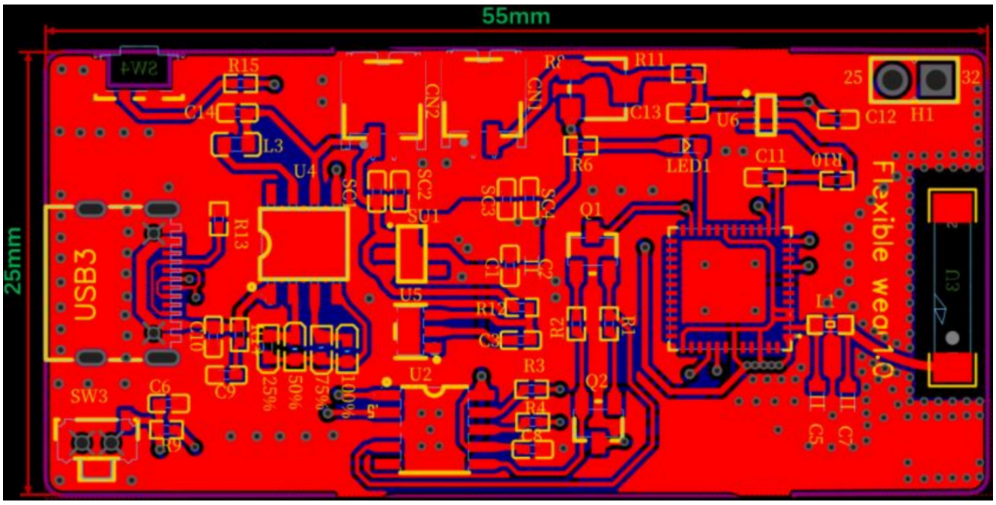
55mm
25mm
USB3
SW3
R15
C14
L3
U4
CN2
SC2
SU1
U5
R12
U2
R3
R4
Q2
R8
R11
C13
U6
C12
H1
25
32
R6
LED1
C11
Q1
R2
R1
C9
C6
25%
50%
75%
100%
C5
C7

(b)

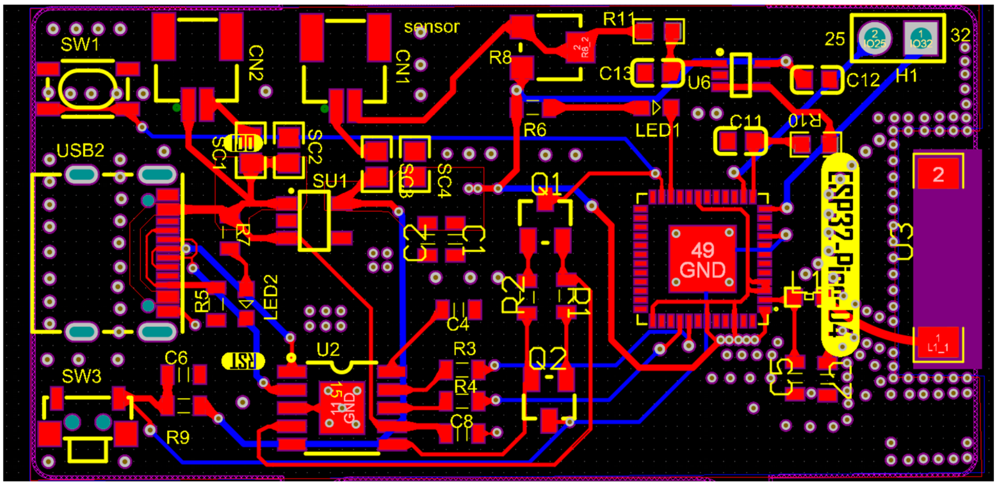
sensor
SW1
CN2
CN1
R8
R11
C13
U6
C12
H1
25
32
R6
LED1
C11
R10
USB2
SC1
SC2
SU1
SC3
SC4
Q1
R7
C2
C1
49
GND
ESP32-Pico-D4
2
U3
R5
LED2
R2
R1
C4
R3
R4
C8
Q2
U2
RST
C6
SW3
R9
C5
C7

(c)

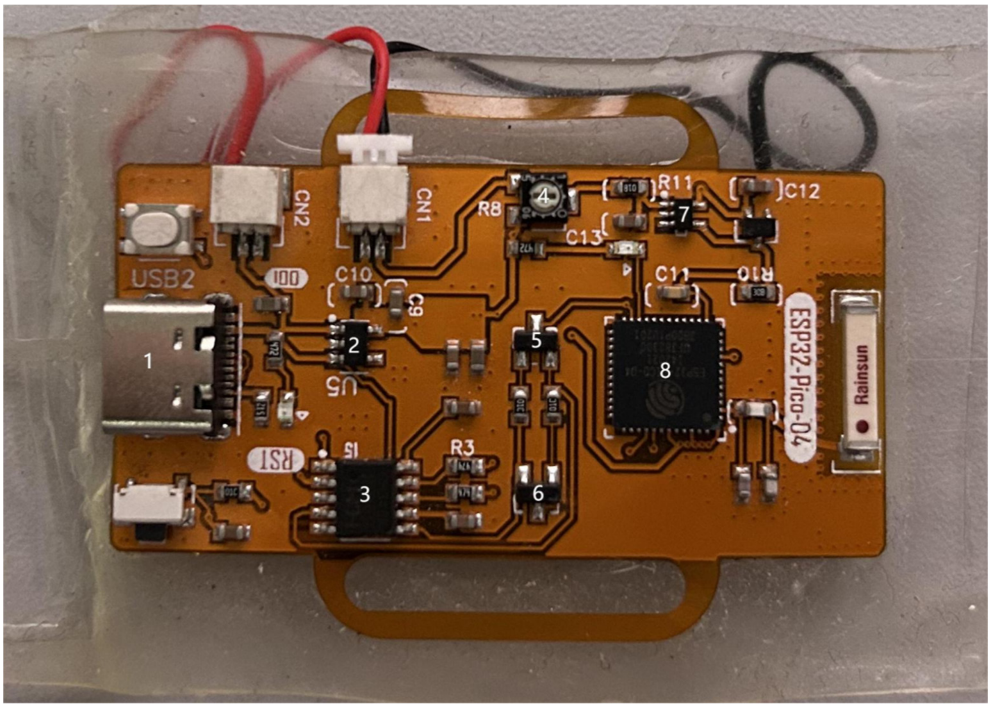
CN2
CN1
USB2
R8
R11
C12
C13
C10
C9
C11
R10
U5
ESP32-Pico-D4
Rainsun
RST
R3
1
2
3
4
5
6
7
8

(d)

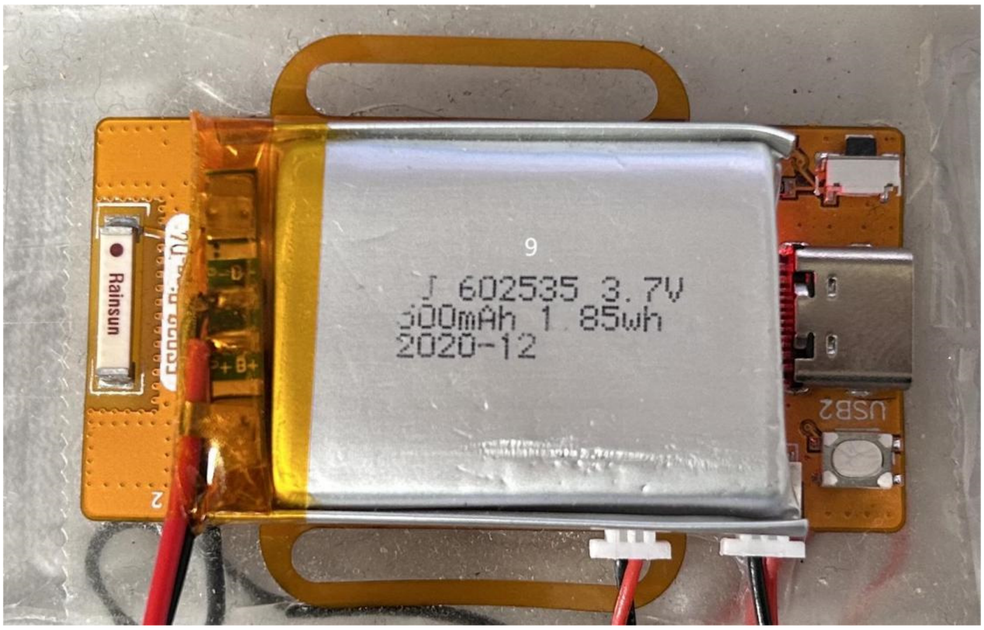

Supplementary Figure S1 | Layouts of the FPC board. (a) Layouts of the FPC board with coppering (b) Layouts of the FPC board without coppering (c) Photos of the FPC board with key components (Supplementary Table S1) labeled (d) Photos of power supply module connecting with FPC board.

Supplementary Table S1 | Key components used in the control electronics. All of the components are commercially off the shelf.

Component designator	Description	Manufacture product number
1	USB connector	TYPE-C 16PIN [illegible]
2	Voltage regulator	SPX3819M5-L-[illegible]
3	USB chip	CH340K
4	Variable resistor	G32AT-B105
5,6	Transistor (NPN）	S8050-J3Y
7	Operational [illegible]	AD8555ARZ
8	MCU and bluetooth module	ESP32-PICO-D 4
9	Power supply module	Li battery

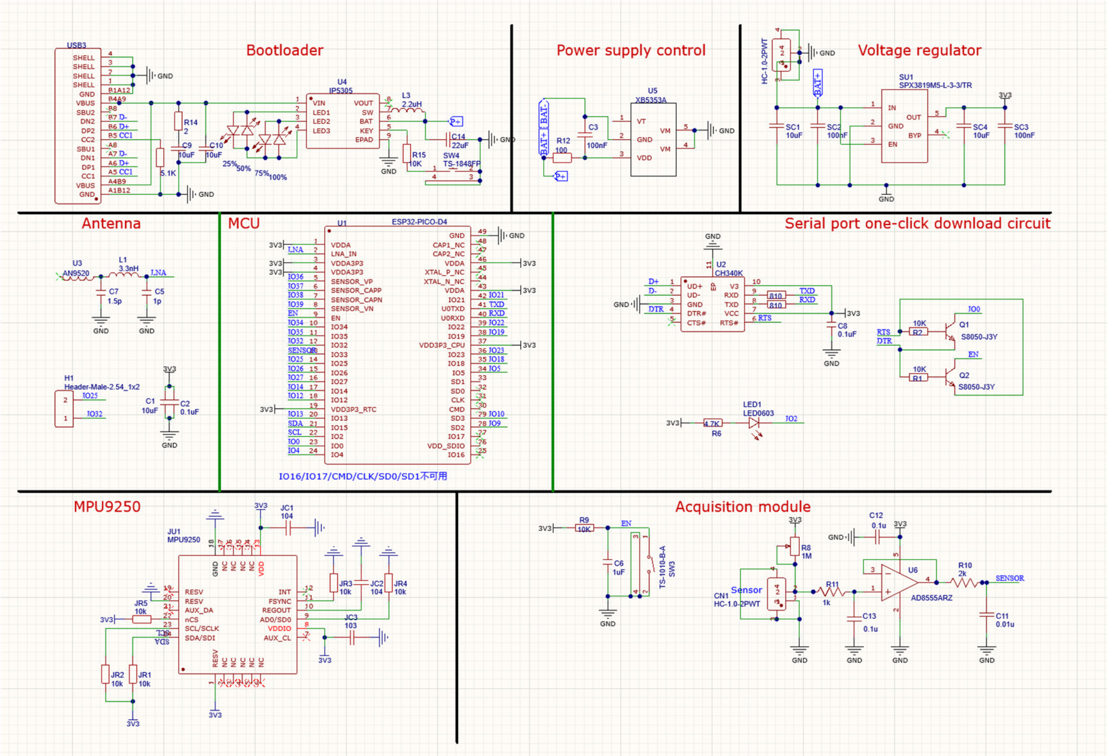

Supplementary Figure S2 | Schematic connections of the analog data acquisition module and bluetooth data transmission module. The analog data acquisition module consists of the AD8555ARZ. The bluetooth data acquisition module consists of a microcontroller (MCU, ESP32-PICO-D4) with on-chip analog-to-digital converter (ADC) and on-chip bluetooth module.
